# Supplementary material for: Development of an epigenetic tetracycline sensor system based on DNA methylation
Source: PLoS One. 2020 May 7;15(5):e0232701. doi: 10.1371/journal.pone.0232701 (PMC7205209; doi:10.1371/journal.pone.0232701)
Supplement: S1 Table — (PDF) [file pone.0232701.s001.pdf]

Supplemental Table 1: Statistical analysis of the readout of the tetracycline memory system used as whole-cell biosensor shown in Figure 6. The indicated p-values refer to the probability of an increase in the number of ON-state cells after induction when compared to the values before induction using a one-tailed two sample t-test (without assuming homogeneity of variance). NS, not significant (p-values larger than 0.05). AI, after induction.

**Tetracycline memory system induced with tetracycline x ng/ml (AI) compared to before induction**	**p-value for fractions of ON-state cells measured with flow cytometry**	
	EGFP	mCherry
AI with 0.1 ng/ml	2.13E-03	NS
AI with 0.2 ng/ml	2.07E-02	1.97E-02
AI with 0.5 ng/ml	7.24E-05	6.96E-03
21 h AI with 0.1 ng/ml	NS	NS
21 h AI with 0.2 ng/ml	4.73E-02	NS
21 h AI with 0.5 ng/ml	7.96E-04	NS
