# Supplementary material for: Development of an epigenetic tetracycline sensor system based on DNA methylation
Source: PLoS One. 2020 May 7;15(5):e0232701. doi: 10.1371/journal.pone.0232701 (PMC7205209; doi:10.1371/journal.pone.0232701)
Supplement: S1 Fig — (PDF) [file pone.0232701.s002.pdf]

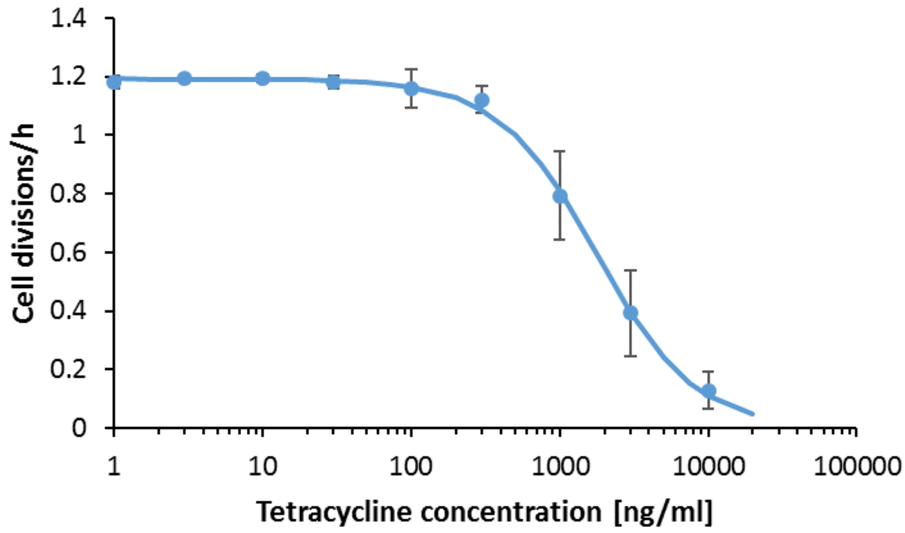

Supplemental Figure 1: Sensitivity of DH5α cells to tetracycline in liquid cultures. Cell division rates were determined in liquid LB culture containing different concentrations of tetracycline during the exponential growth phase at 37 °C. Date are averages of three biological repeats, error bars indicate standard deviations. The line shows a fit to the Hill equation revealing a half-maximal inhibition of cell growth at 1760 ng/ml tetracycline under these conditions.
