# Supplementary material for: Development of an epigenetic tetracycline sensor system based on DNA methylation
Source: PLoS One. 2020 May 7;15(5):e0232701. doi: 10.1371/journal.pone.0232701 (PMC7205209; doi:10.1371/journal.pone.0232701)
Supplement: S2 Fig — (PDF) [file pone.0232701.s003.pdf]

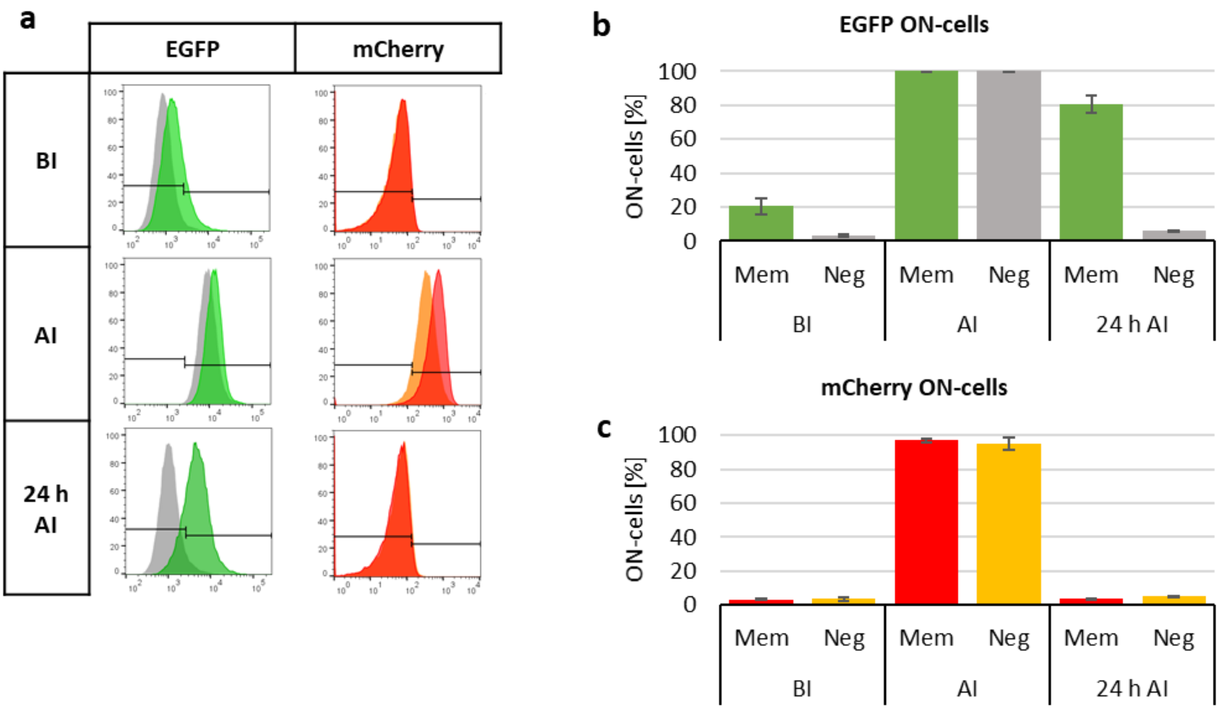

Supplemental Figure 2: Investigation of the tetracycline sensor system at 28 °C with 10 µM $ZnSO_4$. EGFP (green) and mCherry (red) expression was measured by flow cytometry of *E. coli* carrying the TriTet plasmid and either the memory plasmid (Mem) or the negative control memory plasmid with an inactive CcrM mutant (Neg). Cells were cultured without tetracycline (before induction, BI) and with 10 ng/ml tetracycline (after induction, AI). The induced cultures were grown for further 24 h in a medium without tetracycline (24 h AI). a) Histograms of the single cell populations and the applied gates. b) and c) percentage of ON-state cells (ON-cells) for EGFP and mCherry. EGFP signal of the memory system = green bars, EGFP signal of the negative control = gray bars, mCherry signal of the memory system = red bars, mCherry signal of the negative control = orange bars. All averages are based on 3 biological replicates, error bars indicate the SD.
