# Supplementary material for: Development of an epigenetic tetracycline sensor system based on DNA methylation
Source: PLoS One. 2020 May 7;15(5):e0232701. doi: 10.1371/journal.pone.0232701 (PMC7205209; doi:10.1371/journal.pone.0232701)
Supplement: S3 Fig — (PDF) [file pone.0232701.s004.pdf]

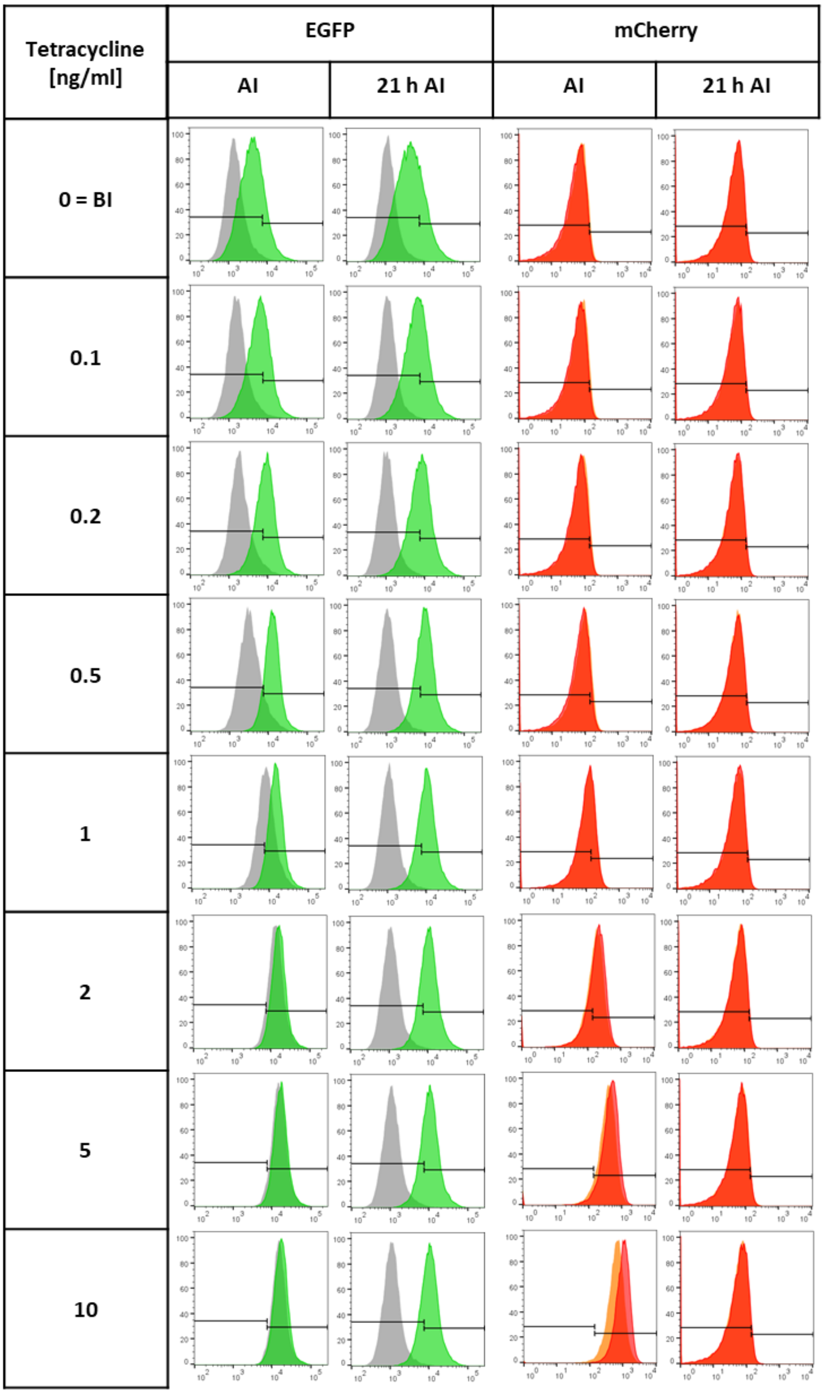

Supplemental Figure 3: ON-state cell analysis of the tetracycline sensor system. Exemplary histograms of the tetracycline memory system (Mem) or the negative control (Neg) of the EGFP (green or gray) and mCherry (red or orange) signals measured by flow cytometry. The gates used to distinguish between ON- and OFF-state cells are indicated. The fluorescence signal is plotted against the number of events and the histograms were normalized to the mode of the number of events. All events on the right side of the gate were defined as ON-state cells. The compiled data are shown in Figure 6.
