# Supplementary material for: Development of an epigenetic tetracycline sensor system based on DNA methylation
Source: PLoS One. 2020 May 7;15(5):e0232701. doi: 10.1371/journal.pone.0232701 (PMC7205209; doi:10.1371/journal.pone.0232701)
Supplement: S1 Text — (PDF) [file pone.0232701.s005.pdf]

Supplemental Text 1: List of all plasmids used in this paper.

Memory plasmid: The DNA sequence is given and annotated in Supplementary Note 1 of Maier et al. (2017). The plasmid map is provided below.

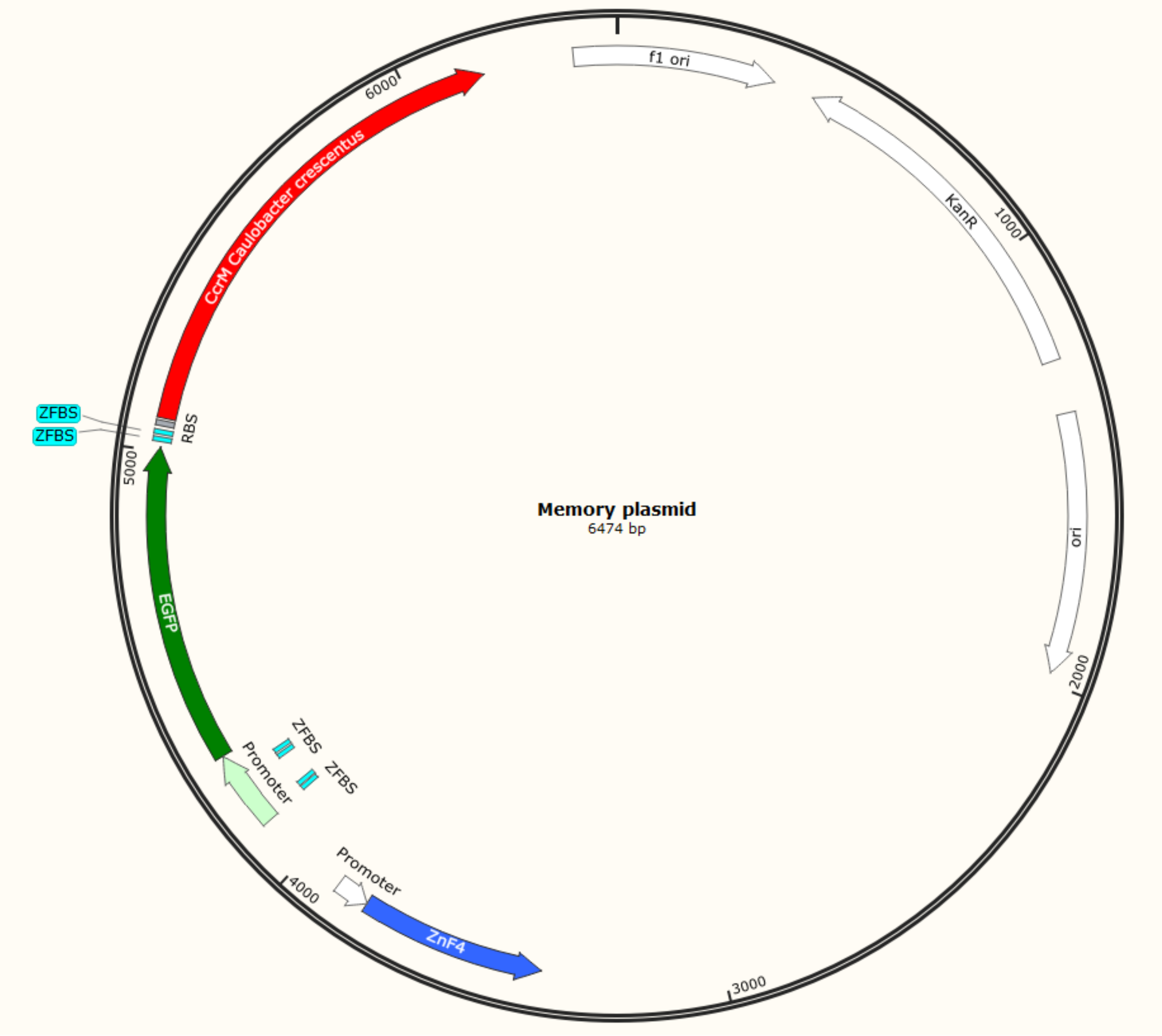

Negative control memory plasmid: The Negative control memory plasmid is identical to the Memory plasmid, except the D31A mutation in the *ccrM* gene, which leads to catalytic inactivation.

Arabinose trigger plasmid: The map of the arabinose trigger plasmid can be found in Maier et al. (2017) Supplementary Figure 7 B.

Tetracycline trigger plasmid: The plasmid map of the tetracycline trigger plasmid can be found in this work (Figure 4 B) and the annotated DNA sequence in Supplemental Text 2.
