# Supplementary material for: Development of an epigenetic tetracycline sensor system based on DNA methylation
Source: PLoS One. 2020 May 7;15(5):e0232701. doi: 10.1371/journal.pone.0232701 (PMC7205209; doi:10.1371/journal.pone.0232701)
Supplement: S2 Text — (PDF) [file pone.0232701.s006.pdf]

Supplemental Text 2: Tetracycline trigger plasmid annotated DNA sequence

```
LOCUS       Exported                5864 bp ds-DNA     linear   SYN 09-JAN-2020
DEFINITION  synthetic linear DNA
ACCESSION   .
VERSION     .
KEYWORDS    TriTet_wRBS_paper (1 - 5864)
SOURCE      synthetic DNA construct
  ORGANISM  synthetic DNA construct
REFERENCE   1  (bases 1 to 5864)
  AUTHORS   Jeltsch Group
  TITLE     Direct Submission
  JOURNAL   Exported Thursday, Jany 9, 2020 from SnapGene 3.3.3
            http://www.snapgene.com
FEATURES             Location/Qualifiers
     source          1..5864
                     /organism="synthetic DNA construct"
                     /mol_type="other DNA"
     CDS             1..439
                     /codon_start=1
                     /note="CcrM Caulobacter crescentus"
                     /translation="MGSSHHHHHHSSGLVPRGSHMASMKFGPETIIHGDCIEQMNALPE
                     KSVDLIFADPPYNLQLGGDLLRPDNSKVDAVDDHWDQFESFAAYDKFTREWLKAARRVL
                     KDDGAIWVIGSYHNIFRVGVAVQDLGFWILNDIVWRKSNPMPNFKGTRFANAHETLIWA
                     SKSQNAKRYTFNYDALKMANDEVQMRSDWTIPLCTGEERIKGADGQKAHPTQKPEALLY
                     RVILSTTKPGDVILDPFFGVGTTGAAAKRLGRKFIGIEREAEYLEHAKARIAKVVPIAP
                     EDLDVMGSKRAEPRVPFGTIVEAGLLSPGDTLYCSKGTHVAKVRPDGSITVGDLSGSIH
                     KIGALVQSAPACNGWTYWHFKTDAGLAPIDVLRAQVRAGMN"
     RBS             465..476
                     /note="strong bacterial ribosome binding site (Elowitz and
                     Leibler, 2000)"
     CDS             483..1190
                     /codon_start=1
                     /product="monomeric derivative of DsRed fluorescent protein
                     (Shaner et al., 2004)"
                     /note="mCherry"
                     /note="mammalian codon-optimized"
                     /translation="MVSKGEEDNMAIIKEFMRFKVHMEGSVNGHEFEIEGEGEGRPYEG
                     TQTAKLKVTKGGPLPFAWDILSPQFMYGSKAYVKHPADIPDYLKLSFPEGFKWERVMNF
                     EDGGVVTVTQDSSLQDGEFIYKVKLRGTNFPSDGPVMQKKTMGWEASSERMYPEDGALK
                     GEIKQRLKLKDGGHYDAEVKTTYKAKKPVQLPGAYNVNIKLDITSHNEDYTIVEQYERA
                     EGRHSTGGMDELYK"
     terminator      1402..1488
                     /gene="Escherichia coli rrnB"
                     /note="rrnB T1 terminator"
                     /note="transcription terminator T1 from the E. coli rrnB
                     gene"
     terminator      1580..1607
                     /note="rrnB T2 terminator"
                     /note="transcription terminator T2 from the E. coli rrnB
```

```
                gene"
promoter        1626..1717
                /gene="bla"
                /note="AmpR promoter"
CDS             1718..2578
                /codon_start=1
                /gene="bla"
                /product="beta-lactamase"
                /note="AmpR"
                /note="confers resistance to ampicillin, carbenicillin, and
                related antibiotics"
                /translation="MSIQHFRVALIPFFAAFCLPVFAHPETLVKVKDAEDQLGARVGYI
                ELDLNSGKILESFRPEERFPMMSTFKVLLCGAVLSRVDAGQEQLGRRIHYSQNDLVEYS
                PVTEKHLTDGMTVRELCSAAITMSDNTAANLLLTTIGGPKELTAFLHNMGDHVTRLDRW
                EPELNEAIPNDERDTTMPVAMATTLRKLLTGELLTLASRQQLIDWMEADKVAGPLLRSA
                LPAGWFIADKSGAGERGSRGIIAALGPDGKPSRIVVIYTTGSQATMDERNRQIAEIGAS
                LIKHW"
rep_origin      2620..3075
                /direction=RIGHT
                /note="f1 ori"
                /note="f1 bacteriophage origin of replication; arrow
                indicates direction of (+) strand synthesis"
rep_origin      3186..3731
                /direction=RIGHT
                /note="p15A ori"
                /note="Plasmids containing the medium-copy-number p15A
                origin of replication can be propagated in E. coli cells
                that contain a second plasmid with the ColE1 origin."
CDS             complement(4428..5051)
                /codon_start=1
                /gene="tetR from transposon Tn10"
                /product="tetracycline repressor TetR"
                /note="TetR"
                /note="TetR binds to the tetracycline operator tetO to
                inhibit transcription. This inhibition can be relieved by
                adding tetracycline or doxycycline."
                /translation="MSRLDKSKVINSALELLNEVGIEGLTTRKLAQKLGVEQPTLYWHV
                KNKRALLDALAIEMLDRHHTHFCPLEGESWQDFLRNNAKSFRCALLSHRDGAKVHLGTR
                PTEKQYETLENQLAFLCQQGFSLENALYALSAVGHFTLGCVLEDQEHQVAKEERETPTT
                DSMPPLLRQAIELFDHQGAEPAFLFGLELIICGLEKQLKCESGS"
promoter        5070..5125
                /gene="tetR"
                /note="tetR/tetA promoters"
                /note="overlapping promoters for bacterial tetR and tetA"
misc_feature    5076..5094
                /note="tet operator O1"
protein_bind    5106..5124
                /gene="tetO"
                /bound_moiety="tetracycline repressor TetR"
                /note="tet operator O2"
                /note="bacterial operator O2 for the tetR and tetA genes"
misc_feature    5126..5129
```

```
                     /note="+1"
     CDS             5160..5864
                     /codon_start=1
                     /note="CcrM Caulobacter crescentus"
                     /translation="MGSSHHHHHHSSGLVPRGSHMASMKFGPETIIHGDCIEQMNALPE
                     KSVDLIFADPPYNLQLGGDLLRPDNSKVDAVDDHWDQFESFAAYDKFTREWLKAARRVL
                     KDDGAIWVIGSYHNIFRVGVAVQDLGFWILNDIVWRKSNPMPNFKGTRFANAHETLIWA
                     SKSQNAKRYTFNYDALKMANDEVQMRSDWTIPLCTGEERIKGADGQKAHPTQKPEALLY
                     RVILSTTKPGDVILDPFFGVGTTGAAAKRLGRKFIGIEREAEYLEHAKARIAKVVPIAP
                     EDLDVMGSKRAEPRVPFGTIVEAGLLSPGDTLYCSKGTHVAKVRPDGSITVGDLSGSIH
                     KIGALVQSAPACNGWTYWHFKTDAGLAPIDVLRAQVRAGMN"
     CDS             5172..5189
                     /codon_start=1
                     /product="6xHis affinity tag"
                     /note="6xHis"
                     /translation="HHHHHH"
     CDS             5199..5216
                     /codon_start=1
                     /product="thrombin recognition and cleavage site"
                     /note="thrombin site"
                     /translation="LVPRGS"
ORIGIN
        1 ctggacccgt tcttcggcgt cggcaccacc ggcgcggccg ccaagcgcct aggccgtaag
       61 ttcatcggca tcgagcgcga ggccgaatac ctcgagcacg ccaaggcccg catcgccaag
      121 gtcgtgccga tcgcgcccga agacctggac gtcatgggct ccaagcgcgc cgagccgcgc
      181 gtgccgttcg gcaccatcgt cgaggccggc ctactgtcgc cgggcgacac gctttactgc
      241 agcaagggca cgcacgtggc caaggtccgt ccggacggct cgatcacggt cggcgacctc
      301 tcgggctcga tccacaagat cggggctctc gttcagagcg cgccggcctg caacggctgg
      361 acctactggc acttcaagac cgacgcgggt ctggcgccga ttgatgtgct gcgagcgcag
      421 gtgcgggcgg ggatgaacta aaggtcgttc atggacttct agagaaagag gagaaatact
      481 agatggtgag caagggcgag gaggataaca tggccatcat caaggagttc atgcgcttca
      541 aggtgcacat ggagggctcc gtgaacggcc acgagttcga gatcgagggc gagggcgagg
      601 gccgccccta cgagggcacc cagaccgcca agctgaaggt gaccaagggt ggccccctgc
      661 ccttcgcctg ggacatcctg tcccctcagt tcatgtacgg ctccaaggcc tacgtgaagc
      721 accccgccga catccccgac tacttgaagc tgtccttccc cgagggcttc aagtgggagc
      781 gcgtgatgaa cttcgaggac ggcggcgtgg tgaccgtgac ccaggactcc tcactgcagg
      841 acggcgagtt catctacaag gtgaagctgc gcggcaccaa cttccccctcc gacggccccg
      901 taatgcagaa gaagaccatg ggctgggagg cctcctccga gcggatgtac cccgaggacg
      961 gcgccctgaa gggcgagatc aagcagaggc tgaagctgaa ggacggcggc cactacgacg
     1021 ctgaggtcaa gaccacctac aaggccaaga agcccgtgca gctgcccggc gcctacaacg
     1081 tcaacatcaa gttggacatc acctcccaca acgaggacta caccatcgtg gaacagtacg
     1141 aacgcgctga gggccgccac tccaccggcg gcatggacga gctgtacaag ctcgagtagg
     1201 gctgttttgg cggatgagag aagattttca gcctgataca gattaaatca gaacgcagaa
     1261 gcggtctgat aaaacagaat ttgcctggcg gcagtagcgc ggtggtccca cctgacccca
     1321 tgccgaactc agaagtgaaa cgccgtagcg ccgatggtag tgtggggtct ccccatgcga
     1381 gagtagggaa ctgccaggca tcaaataaaa cgaaaggctc agtcgaaaga ctgggccttt
     1441 cgttttatct gttgtttgtc ggtgaacgct ctcctgagta ggacaaatcc gccgggagcg
     1501 gatttgaacg ttgcgaagca acggcccgga gggtggcggg caggacgccc gccataaact
     1561 gccaggcatc aaattaagca gaaggccatc ctgacggatg gcctttttgc gtttctacaa
     1621 actcttttgt ttatttttct aaatacattc aaatatgtat ccgctcatga gacaataacc
     1681 ctgataaatg cttcaataat attgaaaaag gaagagtatg agtattcaac atttccgtgt
     1741 cgcccttatt cccttttttg cggcattttg ccttcctgtt tttgctcacc cagaaacgct
```

1801 ggtgaaagta aaagatgctg aagatcagtt gggtgcacga gtgggttaca tcgaactgga
1861 tctcaacagc ggtaagatcc ttgagagttt tcgccccgaa gaacgttttc caatgatgag
1921 cacttttaaa gttctgctat gtggcgcggt attatcccgt gttgacgccg ggcaagagca
1981 actcggtcgc cgcatacact attctcagaa tgacttggtt gagtactcac cagtcacaga
2041 aaagcatctt acggatggca tgacagtaag agaattatgc agtgctgcca taaccatgag
2101 tgataacact gcggccaact tacttctgac aacgatcgga ggaccgaagg agctaaccgc
2161 ttttttgcac aacatggggg atcatgtaac tcgccttgat cgttgggaac cggagctgaa
2221 tgaagccata ccaaacgacg agcgtgacac cacgatgcct gtagcaatgg caacaacgtt
2281 gcgcaaacta ttaactggcg aactacttac tctagcttcc cggcaacaat taatagactg
2341 gatggaggcg gataaagttg caggaccact tctgcgctcg gcccttccgg ctggctggtt
2401 tattgctgat aaatctggag ccggtgagcg tgggtctcgc ggtatcattg cagcactggg
2461 gccagatggt aagccctccc gtatcgtagt tatctacacg acggggagtc aggcaactat
2521 ggatgaacga aatagacaga tcgctgagat aggtgcctca ctgattaagc attggtaact
2581 gtcagaccaa gtttactcat atatacttta gattgattta cgcgccctgt agcggcgcat
2641 taagcgcggc gggtgtggtg gttacgcgca gcgtgaccgc tacacttgcc agcgccctag
2701 cgcccgctcc tttcgctttc ttcccttcct ttctcgccac gttcgccggc tttccccgtc
2761 aagctctaaa tcgggggctc cctttagggt tccgatttag tgctttacgg cacctcgacc
2821 ccaaaaaact tgatttgggt gatggttcac gtagtgggcc atcgccctga tagacggttt
2881 ttcgcccttt gacgttggag tccacgttct ttaatagtgg actcttgttc caaacttgaa
2941 caacactcaa ccctatctcg ggctattctt ttgatttata agggattttg ccgatttcgg
3001 cctattggtt aaaaaatgag ctgatttaac aaaaatttaa cgcgaatttt aacaaaatat
3061 taacgtttac aatttaaaag gatctaggtg aagatccttt ttgataatct catgaccaaa
3121 atcccttaac gtgagttttc gttccactga gcgtcagacc ccgtagaaaa gatcaaagga
3181 tcttcttgag atcgttttgg tctgcgcgta atctcttgct ctgaaaacga aaaaaccgcc
3241 ttgcagggcg gtttttcgaa ggttctctga gctaccaact ctttgaaccg aggtaactgg
3301 cttggaggag cgcagtcacc aaaacttgtc ctttcagttt agccttaacc ggcgcatgac
3361 ttcaagacta actcctctaa atcaattacc agtggctgct gccagtggtg cttttgcatg
3421 tctttccggg ttggactcaa gacgatagtt accggataag gcgcagcggt cggactgaac
3481 ggggggttcg tgcatacagt ccagcttgga gcgaactgcc tacccggaac tgagtgtcag
3541 gcgtggaatg agacaaacgc ggccataaca gcggaatgac accggtaaac cgaaaggcag
3601 gaacaggaga gcgcacgagg gagccgccag gggaaacgc ctggtatctt tatagtcctg
3661 tcgggtttcg ccaccactga tttgagcgtc agatttcgtg atgcttgtca ggggggcgga
3721 gcctatggaa aaacgccagc aacgcggcct ttttacggtt cctggccttt tgctggcctt
3781 ttgctcacat gttctttcct gcgttatccc ctgattctgt ggataaccgt attaccgcct
3841 ttgagtgagc tgataccgct cgccgcagcc gaacgaccga gcgcagcgag tcagtgagcg
3901 aggaagcgga agagcgcctg atgcggtatt ttctccttac gcatctgtgc ggtatttcac
3961 accgcatagg gtcatggctg cgccccgaca cccgccaaca cccgctgacg cgccctgacg
4021 ggcttgtctg ctcccggcat ccgcttacag acaagctgtg accgtctccg ggagctgcat
4081 gtgtcagagg ttttcaccgt catcaccgaa acgcgcgagg cagcaaggag atggcgccca
4141 acagtccccc ggccacgggg cctgccacca tacccacgcc gaaacaagcg ctcatgagcc
4201 cgaagtggcg agcccgatct tccccatcgg tgatgtcggc gatataggcg ccagcaaccg
4261 cacctgtggc gccggtgatg ccggccacga tgcgtccggc gtagaggatc tgctcatgtt
4321 tgacagctta tcatcgatgc ataatgtgcc tgtcaaatgg acgaagcagg gattctgcaa
4381 accctatgct actccgtcaa gccgtcaatt gtctgattcg ttaccaatta agacccactt
4441 tcacatttaa gttgtttttc taatccgcat atgatcaatt caaggccgaa taagaaggct
4501 ggctctgcac cttggtgatc aaataattcg atagcttgtc gtaataatgg cggcatacta
4561 tcagtagtag gtgtttccct ttcttcttta gcgacttgat gctcttgatc ttccaatacg
4621 caacctaaag taaaatgccc cacagcgctg agtgcatata atgcattctc tagtgaaaaa
4681 ccttgttggc ataaaaaggc taattgattt tcgagagttt catactgttt ttctgtaggc
4741 cgtgtaccta aatgtacttt tgctccatcg cgatgactta gtaaagcaca tctaaaactt
4801 ttagcgttat tacgtaaaaa atcttgccag ctttcccctt ctaaagggca aaagtgagta
4861 tggtgcctat ctaacatctc aatggctaag gcgtcgagca aagcccgctt atttttaca

```
4921 tgccaataca atgtaggctg ctctacacct agcttctggg cgagtttacg ggttgttaaa
4981 ccttcgattc cgacctcatt aagcagctct aatgcgctgt taatcacttt acttttatct
5041 aatctagaca tcattaattc ctaatttttg ttgacactct atcgttgata gagttatttt
5101 accactccct atcagtgata gagaaacccg ttttttttggg ctagcaggag gaattcacga
5161 tgggcagcag ccatcatcat catcatcaca gcagcggcct ggtgccgcgc ggcagccata
5221 tggctagcat gaagttcggg ccggaaacca tcatccacgg cgactgcatc gagcagatga
5281 acgccctgcc cgagaagtcg gtcgacctga tcttcgccga tccgccctat aacctgcagt
5341 tgggcgggga cctcctgcgt cccgacaatt ccaaggtcga cgcggtcgac gaccactggg
5401 accagttcga gagcttcgcc gcctacgaca agttcacccg cgagtggctg aaggccgccc
5461 gccgcgttct gaaggacgac ggcgcgatct gggtgatcgg cagctatcac aacatcttcc
5521 gcgtcggcgt ggccgtgcag gacctgggct tctggatcct caacgacatc gtctggcgca
5581 agtccaaccc gatgcccaac ttcaagggca cccgcttcgc caacgctcac gagaccctga
5641 tctgggcctc caagagccag aacgccaagc gctacacctt caattacgac gccctgaaga
5701 tggccaatga cgaggtgcag atgcgctcgg actggaccat cccgctgtgc accggcgagg
5761 agcgcatcaa gggcgccgac ggccagaaag cccacccgac ccagaagccc gaggccctgc
5821 tctaccgcgt catcctgtcg acgacgaagc cgggcgacgt gatc
//
```
